# Electroconvulsive therapy-specific volume changes in nuclei of the amygdala and their relationship to long-term anxiety improvement in depression

**DOI:** 10.1038/s41380-024-02874-1

**Published:** 2024-12-16

**Authors:** Yuzuki Ishikawa, Naoya Oishi, Yusuke Kyuragi, Momoko Hatakoshi, Jinichi Hirano, Takamasa Noda, Yujiro Yoshihara, Yuri Ito, Jun Miyata, Kiyotaka Nemoto, Yoshihisa Fujita, Hiroyuki Igarashi, Kento Takahashi, Shingo Murakami, Hiroyuki Kanno, Yudai Izumi, Akihiro Takamiya, Junya Matsumoto, Fumitoshi Kodaka, Kazuyuki Nakagome, Masaru Mimura, Toshiya Murai, Taro Suwa

**Affiliations:** 1 Department of Psychiatry, Graduate School of Medicine, Kyoto University, Kyoto, Japan; 2 Human Brain Research Center, Graduate School of Medicine, Kyoto University, Kyoto, Japan; 3 Department of Neuropsychiatry, Keio University School of Medicine, Tokyo, Japan; 4 Department of Psychiatry, National Center of Neurology and Psychiatry, Tokyo, Japan; 5 Department of Psychiatry, Aichi Medical University, Aichi, Japan; 6 Department of Psychiatry, Institute of Medicine, University of Tsukuba, Ibaraki, Japan; 7 Neuropsychiatry, Department of Neurosciences, Leuven Brain Institute, KU Leuven, Leuven, Belgium; 8 Hills Joint Research Laboratory for Future Preventive Medicine and Wellness, Keio University School of Medicine, Tokyo, Japan; 9 Department of Pathology of Mental Diseases, National Institute of Mental Health, National Center of Neurology and Psychiatry, Tokyo, Japan; 10 Department of Psychiatry, The Jikei University School of Medicine, Tokyo, Japan; 11 National Center of Neurology and Psychiatry, Tokyo, Japan

**Keywords:** Neuroscience, Depression

## Abstract

Electroconvulsive therapy (ECT) is one of the most effective treatments for depression. ECT induces volume changes in the amygdala, a key center of anxiety. However, the clinical relevance of ECT-induced changes in amygdala volume remains uncertain. We hypothesized that nuclei-specific amygdala volumes and anxiety symptoms in depression could explain the clinical correlates of ECT-induced volume changes. To test this hypothesis, we enrolled patients with depression who underwent ECT (N = 20) in this multicenter observational study and collected MRI data at three time points: before and after treatment and a 6-month follow-up. Patients who received medication (N = 52), cognitive behavioral therapy (N = 63), or transcranial magnetic stimulation (N = 20), and healthy participants (N = 147) were included for comparison. Amygdala nuclei were identified using FreeSurfer and clustered into three subdivisions to enhance reliability and interpretability. Anxiety symptoms were quantified using the anxiety factor scores derived from the Hamilton Depression Rating Scale. Before treatment, basolateral and basomedial subdivisions of the right amygdala were smaller than those of healthy controls. The volumes of the amygdala subdivisions increased after ECT and decreased during the follow-up period, but the volumes at 6-month follow-up were larger than those observed before treatment. These volume changes were specific to ECT. Long-term volume changes in the right basomedial amygdala correlated with improvements in anxiety symptoms. Baseline volumes in the right basolateral amygdala correlated with long-term improvements in anxiety symptoms. These findings demonstrate that clinical correlates of ECT-induced amygdala volume changes are existent, but in a nucleus and symptom-specific manner.

## Introduction

Depression is a neuropsychiatric disorder characterized by persistent feelings of sadness, loss of interest, and other psychosocial dysfunctions [1]. Its heterogeneous symptom profiles and variability in individual responses to treatment make successful treatment procedures difficult, with approximately one-third of patients resistant to antidepressants [2, 3]. Electroconvulsive therapy (ECT) is a highly effective treatment for treatment-resistant depression, with reported remission rates of 60–80% [4, 5]. This remission rate is higher than that of other depression treatment modalities, such as medication, cognitive behavioral therapy (CBT), and transcranial magnetic stimulation (TMS) [2, 6–8]. Despite its outstanding effectiveness, the neural mechanisms underlying the action of ECT are not fully understood [9]. Furthermore, it remains unclear which neurobiological changes induced by ECT indicate the therapeutic effects. Identifying the neural substrates associated with the therapeutic effects of ECT is crucial for understanding depression mechanisms, and it may be the key to predicting treatment responsiveness. Consequently, previous studies have explored the neurobiological correlates of ECT at various levels [10–12].

One established finding from studies on ECT is the extensive changes in brain volume, especially in limbic structures such as the hippocampus and amygdala [13–16]. Studies on the hippocampus suggest that its volume increase is associated with changes in the markers of neural plasticity and synaptic structures, rather than being a result of edema or angiogenesis [17–19]. Nevertheless, conflicting evidence exists regarding the relationship between volume increases and therapeutic effects [15, 20–22], and a meta-analysis on the hippocampus reported no such associations [13]. Similarly, previous findings on the relationship between volume changes in the amygdala and clinical outcomes have been inconsistent [23–26], complicating our understanding of its role in the therapeutic effects of ECT. Given the crucial role of the amygdala in anxiety, a core symptom of depression [27–31], investigating the association between amygdala volume changes and the therapeutic effects of ECT is crucial for a comprehensive understanding of depression.

The amygdala consists of structurally and functionally heterogeneous nuclei [30, 32], which could explain the conflicting findings regarding its association with therapeutic effects. The basolateral complex of the amygdala integrates sensory inputs from the cortical regions and the thalamus, while the central nucleus of the amygdala relays information to modulate behavioral, physiological, and affective responses [30, 33]. Thus, numerous animal studies have focused on the nucleus-specific characteristics of the amygdala [34–36]. Additionally, several human studies have compared the volumes of amygdala nuclei in patients with depression to those in healthy individuals [37–40]. These studies have suggested that therapeutic effects may not be evident when considering the amygdala as a singular entity but could be apparent at the nucleus level. Although there are several longitudinal studies on ECT-induced amygdala volume changes [23, 25, 41], only one study has examined the clinical associations of amygdala volume changes at the nucleus level [26], possibly ignoring nucleus-level heterogeneity.

Previous studies’ conflicting findings on the relationship with clinical outcomes may also stem from depression’s symptom-level heterogeneity. Depressive symptoms are typically assessed using the total score of the Hamilton Depression Rating Scale (HAMD), Montgomery Asberg Depression Rating Scale (MADRS), or Beck Depression Inventory (BDI) [42–44]. These rating scales reflect multiple symptom dimensions such as anhedonia and anxiety, which may hinder the understanding of treatment effects at the symptom level [45, 46]. Both human- and non-human studies have established the involvement of the amygdala in anxiety [27–31]. Furthermore, in patients with depression, prelimbic functional connectivity features, particularly involving the amygdala, correlate with anxiety and sleep disturbances. However, these features do not correlate with anhedonia or psychomotor retardation [47]. These findings highlight the importance of further investigation into how changes in amygdala volume induced by ECT relate to anxiety symptoms and the potential as a predictive biomarker for anxiety improvements.

Furthermore, comparing ECT with other treatment modalities is crucial to better understanding the relationship between volume changes and the therapeutic effects. Various depression treatments may induce common neuroplastic changes such as synapse formation, increased neurotropic factors, or neurogenesis [48, 49]. In contrast, the therapeutic effects of ECT are more rapid than those of medications, suggesting that distinct neurobiological mechanisms underlie each treatment [50]. Given these findings and the distinctive therapeutic effects of ECT, there is a clear need for cross-treatment comparisons. Previous studies have indicated that ECT-induced volume increases are transient and followed by subsequent decreases [51, 52], highlighting the importance of examining long-term changes. A previous study compared ECT with treatment as usual such as medication, structured psychotherapy, and case management, and found that the amygdala volume increase immediately after the completion of treatment was specific to ECT [41]. However, findings on long-term volume changes across various treatment modalities are largely lacking, limiting our understanding of the long-term neural correlates of different treatment approaches.

Therefore, this observational study aims to examine short- and long-term volume changes in the nuclei of the amygdala following ECT and other treatment modalities. Furthermore, the study aims to identify clinical correlates of the ECT-induced volume changes in the amygdala nuclei by focusing on their association with anxiety symptoms. To achieve our objectives, we included patients with depression who received ECT, medication, CBT, and TMS, as well as healthy participants. MRI images were acquired at three time points: pre-treatment, post-treatment, and at the 6-month follow-up. We hypothesized that (1) volume changes in the amygdala nuclei induced by treatment are specific to ECT across both short- and long-term durations, (2) a significant association exists between amygdala volumes and the effectiveness of treatment, particularly in its nuclei and anxiety symptoms, and (3) baseline volumes in the amygdala nuclei are predictive of subsequent improvements in anxiety symptoms.

## Materials and methods

### Participants and study design

Participants were recruited for the Longitudinal MRI study Identifying the Neural Substrates of Remission/Recovery in Mood Disorders (L/R Study), a constituent of the Brain MINDS/Beyond project [53]. The study included 155 patients with depression (aged 20–80 years; 74 men, 81 women) and 147 healthy participants (aged 20–78 years; 59 men, 88 women) sourced from four sites. The demographic and clinical data of the participants are presented in Table 1.

**Table 1:** Demographics and clinical characteristics.

Characteristic	Study group				Study group	
	ECT	Other treatment group				
		Medication	CBT	TMS	Depression	Healthy controls
N	20	52	63	20	155	147
Sex (M/F)	7/13	31/21	25/38	11/9	74/81	59/88
Age, mean (SD), years	55.1 (17.1)	47.2 (15.6)	39.1 (13.1)*	43.4 (14.9)*	44.5 (15.6)	42.0 (13.1)
	Clinical score				Clinical score	
Pre HAMD-17	25.0 (6.3)	14.7 (5.8)*	14.7 (4.4)*	15.8 (4.9)*	16.1 (6.2)	-
Post HAMD-17	7.8 (5.6)	8.3 (6.7)	8.4 (6.0)	10.3 (6.9)	8.6 (6.3)	-
Follow HAMD-17	6.7 (7.2)	7.1 (7.5)	7.3 (5.3)	9.5 (6.4)	7.4 (6.5)	-
Pre anxiety factor score	6.15 (2.22)	3.79 (1.74)*	3.72 (1.41)*	3.47 (1.79)*	4.03 (1.87)	-
Post anxiety factor score	1.72 (1.38)	2.30 (1.99)	2.25 (1.66)	2.09 (1.40)	2.18 (1.71)	-
6MA anxiety factor score	1.36 (1.61)	1.94 (2.10)	1.88 (1.57)	2.04 (1.93)	1.85 (1.81)	-
Remission at Post (yes/no)	11/9	26/26	31/32	10/10	78/77	-
Remission at 6MA (y/n)	14/6	27/25	31/32	8/12	80/75	-
Response at Post (y/n)	16/4	35/17	36/27	11/9	98/57	-
Relapse at 6MA (y/n)	2/9	1/25	8/23	3/7	14/64	-

Remission: HAMD-17 < 8. Response: ≧50% HAMD-17 improvement.

*HAMD-17* Hamilton 17-item Depression Rating Scale, *ECT* electroconvulsive therapy.

*P < 0.05 in comparison to the ECT group.

All patients were scheduled to receive either antidepressant medication, ECT, CBT, or TMS as part of their regular treatment based on the discretion of their attending psychiatrist at the time of recruitment. All patients met the criteria for major depressive disorder or persistent depressive disorder according to the DSM-5 [1]. The key exclusion criteria encompassed substance use or alcohol misuse within the past 2 years, current suicidal ideation or suicide attempts in the past, current manic episodes or diagnosis of dementia, unstable medical condition, or other severe illnesses.

**Fig. 1:**
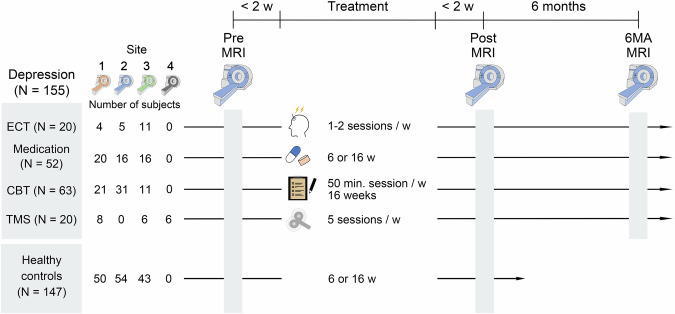
Schematic overview of the study design. Data acquisition was conducted across four institutions. All treatment groups underwent MRI scanning and clinical assessments three times: once each within 2-week periods before and after treatment (Pre and Post), and once in 6 months after treatment (6MA). Patients were assigned into four treatment groups based on the discretion of their attending psychiatrist. Healthy participants underwent MRI scanning and clinical assessments two times at intervals of 6 or 16 weeks. ECT electroconvulsive therapy, CBT cognitive behavioral therapy, TMS transcranial magnetic stimulation.

All patients underwent MRI scanning and clinical assessments three times: within 2-weeks before (Pre) and after (Post) treatment, and 6 months after treatment (6MA). Healthy participants underwent MRI and clinical assessments twice at intervals of 6 or 16 weeks. Depressive symptoms were evaluated at each time point using the Hamilton 17-item Depression Rating Scale (HAMD-17) [42]. Inclusion in the depression group required a HAMD-17 score of $\ge$ 8 at the time of the recruitment. All the participants provided written informed consent to participate in this study. An overview of the study timeline is depicted in Fig. 1.

### Treatment

The attending psychiatrist assigned patients to one of four treatment groups based on their discretion. The ECT group (N = 20; aged 24–80 years; 7 men and 13 women) received ECT sessions twice per week except for cases of severe physical illnesses (see Supplementary Results). ECT treatment was performed using a brief pulse square-wave device (Thymatron System IV; Somatics, LLC, Venice, FL, USA). All patients in the ECT group met the criteria for ECT according to the American Psychiatric Association task force guidelines [54]. Specifically, patients must be classified as treatment-resistant, urgent, or intolerant to pharmacotherapy. Treatment resistance was defined as the failure of two or more antidepressants administered at adequate dosages and for sufficient durations [54]. Treatment sessions were continued if the patient did not experience symptom relief. Electrode placement was either bitemporal (N = 9), bifrontal (N = 9), or right unilateral (N = 2). During the treatment course, two patients switched from bitemporal to bifrontal, and one patient switched from right unilateral to bifrontal. Anesthesia was induced using either propofol (1–2 mg/kg body weight, N = 15), thiopental sodium (2–4 mg/kg body weight, N = 3), ketamine (60 mg, N = 1), or sevoflurane (7.2–8%, N = 4). Decisions on the ECT treatment regimen were based on clinical assessments (e.g., responsiveness to previous ECT sessions, severity of depression). Detailed parameter settings are provided in Supplementary Methods and Table S1. The medication group (N = 52; aged 21–78 years, 31 men and 21 women) included cases involving add-on therapy, switch medications, and treatment-naïve cases. However, the core group consisted of add-on cases in which a single antidepressant was ineffective. The duration of treatment for the medication group was either 6 or 16 weeks. The CBT group (N = 63; age range: 20–79 years; 25 men, 38 women) underwent 16 consecutive 50-min sessions per week (16 weeks in total). The TMS group (N = 20; age: 20–74 years; 11 men, 9 women) underwent TMS sessions up to five times per week using a Neurostar TMS Therapy System (Malvern, PA, USA) with a figure-of-eight stimulation coil. Detailed parameter settings for TMS are provided in Supplementary Table S2. The concomitant use of antidepressants, mood stabilizers, antipsychotics, and anxiolytics was not prohibited in any treatment group.

### MRI acquisition

MRI data were acquired using either one of six scanners installed at four different sites. All the images were acquired at a field strength of 3T. At Site 1 (Keio University, N = 103), 3D T1-weighted brain images were collected using either a GE Discovery MR750 (GE HealthCare, Illinois, USA) or SIGNA HDxt (GE Healthcare). At sites 2 (Kyoto University, N = 106), 3 (National Center Hospital, National Center of Neurology and Psychiatry, N = 87), and 4 (The Jikei University, N = 6), brain images were obtained using three-dimensional magnetization-prepared rapid acquisition with a gradient echo, utilizing a MAGNETOM Verio, MAGNETOM Skyra or MAGNETOM Skyra fit scanner (all from Siemens Healthineers, Erlangen, Germany). Detailed scanning parameters and correspondence between the treatment groups and scanners are provided in Supplementary Table S3.

### MRI preprocessing

The structural images underwent bias-field correction using Advanced Normalization Tools ver. 2.4.4. [55] and were processed using the longitudinal standard pipeline [56] in FreeSurfer ver. 7.3.2. This pipeline creates an unbiased within-subject template space and image using a robust, inverse-consistent registration based on outputs from the cross-sectional pipeline [57]. Subsequently, the preprocessing steps were initialized with the new template image to enhance reliability and statistical power. Following preprocessing, the intracranial volumes (ICV) were extracted from the recon-all pipeline output and used for subsequent analyses.

### Amygdala segmentation

The T1 longitudinal amygdala segmentation module in FreeSurfer ver. 7.3.2. [58, 59] was applied to bias-field-corrected, normalized, and skull-stripped images generated from the FreeSurfer longitudinal pipeline. This module employs a probabilistic atlas built using ultra-high-resolution ex vivo MRI data and image intensity to delineate nine amygdala nuclei [59]. The longitudinal segmentation module provides a generative model-based joint segmentation of nuclei across multiple time points to yield more reliable subregional volumes [58, 59]. The amygdala nuclei were visually inspected for quality assurance; however, no manual modifications were made to avoid introducing external bias.

### Amygdala nuclei clustering

**Fig. 2:**
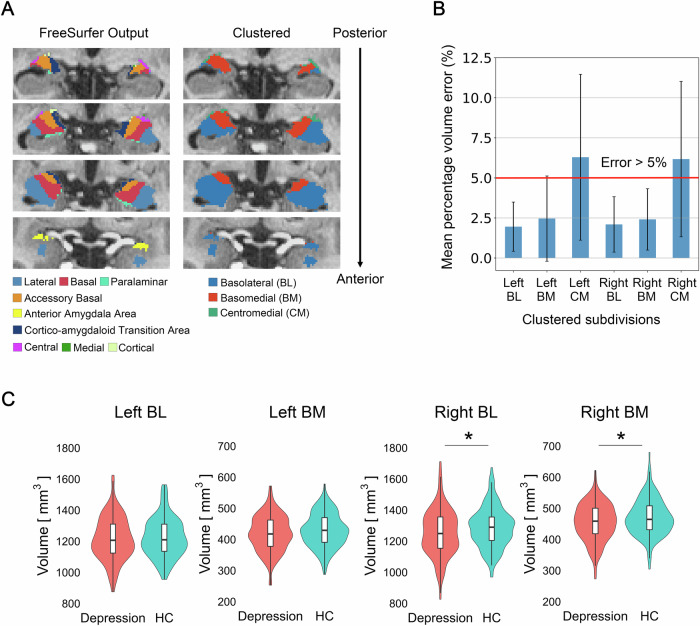
Amygdala subdivisions and their baseline volumes. **A** A representative image of the amygdala parcellation. (Left) Nine nuclei are identified from the FreeSurfer output. (Right) Three clustered subdivisions. The basolateral subdivision consists of the lateral, basal, paralaminar nucleus, and the anterior amygdaloid area. The basomedial subdivision is comprised of the accessory basal nucleus and the cortico-amygdaloid transition area. The centromedial subdivision consists of the central, cortical, and medial nucleus. **B** Test-retest reliability of the clustered subdivisions. The bilateral centromedial subdivisions showed a mean percentage volume error of >5% and were excluded from further analyses. **C** Baseline amygdala subdivision volumes of patients with depression and healthy controls. BL Basolateral, BM Basomedial, CM Centromedial, HC Healthy controls. *: *P* < 0.05 Bonferroni corrected.

The FreeSurfer segmentation module exhibits high test-retest reliability for most nuclei. However, some nuclei show relatively low reliability, which is critical for making longitudinal comparisons [60]. Additionally, these nuclei cannot be directly compared with the typical partitioning in prior human studies, where the amygdala was divided into two or three subdivisions [32, 61, 62]. To address these problems, we clustered the nine amygdala nuclei into three subdivisions based on their anatomical plausibility and proximity [30, 32, 59, 63] (Fig. 2A). The first subdivision consists of the lateral nucleus, basal nucleus, paralaminar nucleus, and anterior amygdala, collectively referred to as the basolateral (BL) subdivision. The second subdivision comprises the accessory basal nucleus and cortico-amygdaloid transition area, referred to as the basomedial (BM) subdivision. The third subdivision consists of the central, medial, and cortical nucleus, collectively referred to as the centromedial (CM) subdivision.

### Test-retest reliability of amygdala subdivision volumes

To assess whether the test-retest reliability of the clustered amygdala subdivisions is adequate for longitudinal comparison, we quantified the test-retest reliability as the percentage volume error (ε) between Pre and Post volume measurements of healthy controls [64] using the following formula:

1 $${{\rm{\varepsilon }}}=100\times \frac{{{|}}{V}_{{Pre}}-{V}_{{Post}}{{|}}}{({V}_{{Pre}}+{V}_{{Post}})/2}$$

We established that a percentage volume error of less than 5% was sufficient for the longitudinal comparison [60]. Therefore, subdivisions with errors greater than 5% were excluded from further analysis.

### Demographics and clinical data

The mean and standard deviation for continuous variables, as well as the number and ratio of categorical variables were calculated using Python 3.8. *t*-tests and chi-squared tests were used to compare the differences between the ECT group and other treatment groups, and between the depression group and the healthy control group. Statistical significance was set to *P* < 0.05.

### Harmonizing scanner differences

Cross-sectional and longitudinal ComBat harmonization [65–67] were used to adjust volumes for cross-scanner differences. ComBat is a batch-effect correction tool designed to remove inter-scanner technical variability while maintaining inter-scanner biological variability [67]. Age, sex, ICV, and the clinical outcomes of interest were integrated into the batch-effect estimation to retain the effects of these variables. ComBat was performed separately using R 4.3.1 for each statistical analysis, and the same covariates were used for each analysis.

### Comparison of pre-treatment volume between patients with depression and healthy controls

The baseline volumes of amygdala subdivisions in patients with depression were compared with those of healthy controls. To elucidate group differences, an analysis of covariance (ANCOVA) incorporating age, sex, and ICV as covariates was performed using R version 4.3.1. Statistical significance was set at *P* < 0.05, with the Bonferroni correction adjusting for side (left, right) and subdivision (BL and BM).

### Longitudinal volume change

First, repeated measures ANCOVA, with ICV as a covariate, was performed separately within each treatment group to examine whether there was a significant volume change between any of the time points. For significant results, post-hoc tests were performed using linear mixed-effects models with time and ICV as fixed effects and individuals as random intercepts to identify the significant changes between specific timepoint pairs (Pre and Post, Post and 6MA, and Pre and 6MA). The mean percentage of the volume change between each time point was calculated to illustrate the effect size. Spearman’s rank correlation analysis was performed to examine the relationship between volume changes in the amygdala subdivisions and the number of ECT sessions. Statistical significance was set at *P* < 0.05 with the Bonferroni correction adjusting for side (left, right) and subdivision (BL and BM). All statistical analyses were performed using R version 4.3.1.

### Association with anxiety factor score

Factor loadings of the anxiety component in the HAMD-17, derived from a large-scale meta-analysis of more than 2600 patients [45], were used to investigate the association between amygdala volume and anxiety symptoms in the ECT group. These loadings reflect the degree to which items co-occurred under the same factor in previous studies and are provided in the Supplementary Methods. Confirmatory factor analysis of these factors showed a moderate-to-good fit, indicating their replicability [68]. Partial correlations between baseline volume and anxiety factor scores, with age, sex, and ICV considered as covariates, were calculated.

Changes in volume and anxiety factor scores were defined as the difference between two timepoints:

2 $$\Delta {{{\rm{Volume}}}}_{{{\rm{tp}}}1\to {{\rm{tp}}}2}={{{\rm{Volume}}}}_{{{\rm{tp}}}2}-{{{\rm{Volume}}}}_{{{\rm{tp}}}1}$$

3 $$\Delta {{\rm{Anxiety}}}\; {{\rm{factor}}}\; {{\rm{score}}}_{{{\rm{tp}}}1\to {{\rm{tp}}}2} = 	 \,{{{\rm{Anxiety}}}\; {{\rm{factor}}}\; {{\rm{score}}}}_{{{\rm{tp}}}2}\\ 	 -{{{\rm{Anxiety}}}\; {{\rm{factor}}}\; {{\rm{score}}}}_{{{\rm{tp}}}1}$$

Partial correlation between Δvolume and Δanxiety factor score with age, sex, and ICV as covariates was calculated for each timepoint. Additionally, partial correlation analysis between the baseline volume and Δanxiety factor score was performed to examine whether baseline volumes correlated with improvements in anxiety symptoms.

Correlation analyses were also performed for the overall HAMD-17 scores to determine whether the anxiety component exhibited a greater correlation compared to the total HAMD-17 score. Statistical significance was set at *P* < 0.05 with the Bonferroni correction adjusting for side (left, right) and subdivision (BL and BM). All statistical analyses in this section were performed using R version 4.3.1.

## Results

### Clinical characteristics and treatment outcomes

The overall post-treatment remission rate was 50.3% and did not differ across the treatment modalities (Table 1). At the 6MA, the ECT group had the highest remission rate of 70%; however, this difference was not statistically significant compared with that of any other treatment group. The ECT group and each non-ECT treatment group did not differ in sex ratio, but they significantly differed in age, except for the medication group. The baseline HAMD-17 and anxiety factor scores were significantly different between the ECT and non-ECT treatment groups.

### Amygdala nuclei clustering

The test-retest reliability of volumetrics increased after clustering the nine nuclei from the FreeSurfer output into three subdivisions (Fig. 2A, B, Supplementary Fig. S1). However, the bilateral CM subdivisions showed >5% volume error and were excluded from further analyses (Fig. 2B).

### Baseline amygdala subdivision volumes

Baseline amygdala volumes in patients with depression were significantly lower in the right BL subdivision (*F*[1, 297] = 6.99, *P* = 0.009, *d* = 0.27) and the right BM subdivision (*F*[1, 297] = 8.12, *P* = 0.005, *d* = 0.29) compared with those of healthy controls (Fig. 2C, Supplementary Table S4). None of the amygdala subdivisions exhibited significant partial correlations with the total HAMD-17 or anxiety factor scores after adjusting for age, sex, and ICV.

### Longitudinal volume changes in amygdala subdivisions

**Fig. 3:**
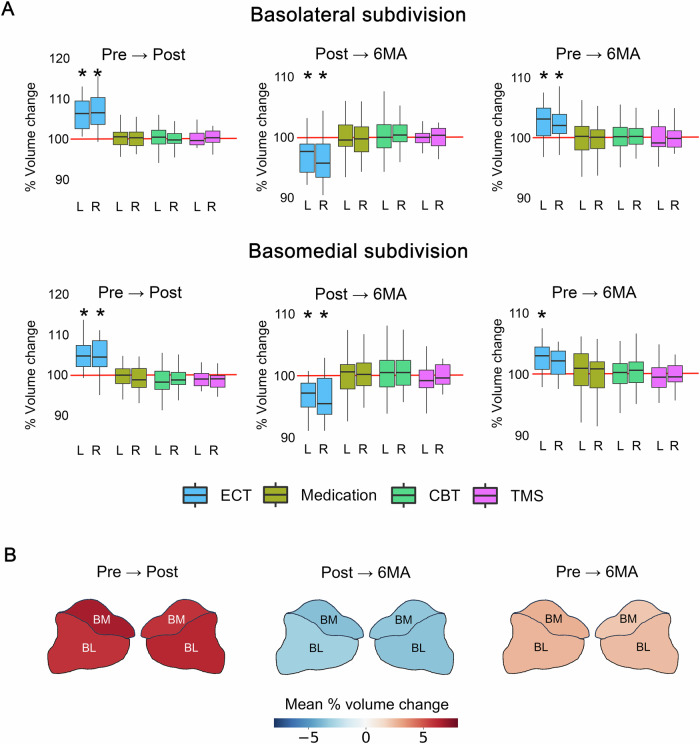
Longitudinal changes in amygdala subdivision volumes. **A** Percentage volume changes of the amygdala subdivisions for each group. **B** Heatmaps depicting mean percentage volume changes for the ECT group. ECT electroconvulsive therapy, CBT cognitive behavioral therapy, TMS transcranial magnetic stimulation, BL Basolateral, BM Basomedial. *: *P* < 0.05 Bonferroni corrected.

The ECT group demonstrated significant Pre-to-Post volume increases in both the BL (Left: β = 0.49, *SE* = 0.08, *P* < 0.001, μ = 5.9%; Right: β = 0.48, *SE* = 0.08, *P* < 0.001, μ = 6.2%) and BM (Left: β = 0.40, *SE* = 0.05, *P* < 0.001, μ = 6.7%; Right: β = 0.35, *SE* = 0.07, *P* < 0.001, μ = 5.8%) subdivisions, followed by significant Post-to-6MA volume decreases in both the BL (Left: β = −0.25, *SE* = .07, *P* = 0.003, μ = −3.0%; Right: β = −0.26, *SE* = 0.07, *P* = 0.001, μ = −3.4%) and BM (Left: β = −0.23, *SE* = 0.04, *P* < 0.001, μ = −3.6%; Right: β = −0.20, *SE* = 0.05, *P* = 0.001, μ = −3.4%) subdivisions (Fig. 3, Supplementary Figs. S2, 3, Table S6).

The left BL, BM, and right BL subdivisions showed significantly larger volumes at 6MA than those at pre-treatment (Left BL: β = 0.12, *SE* = 0.04, *P* = 0.003, μ = 2.6%; Left BM: β = 0.08, *SE* = 0.02, *P* = 0.002, μ = 2.8%; Right BL: β = 0.09, *SE* = 0.02, *P* = 0.001, μ = 2.4%) (Fig. 3, Supplementary Figs. S2, 3, Table S6). None of the other treatment groups exhibited significant volume changes between any of the timepoints (Fig. 3, Supplementary Figs. S2, 3, Table S6). Additionally, ECT-specific volume changes persisted even after adjusting for age and baseline anxiety factor scores (Supplementary Figs. S4, 5 and Tables S7, 8). There was no association between the number of ECT sessions and volume changes (Supplementary Tables S1 and S9). There were no significant differences in volume changes between remitters and non-remitters at Post (Supplementary Fig. S6).

### Association between volume changes in the amygdala subdivisions and anxiety severity

**Fig. 4:**
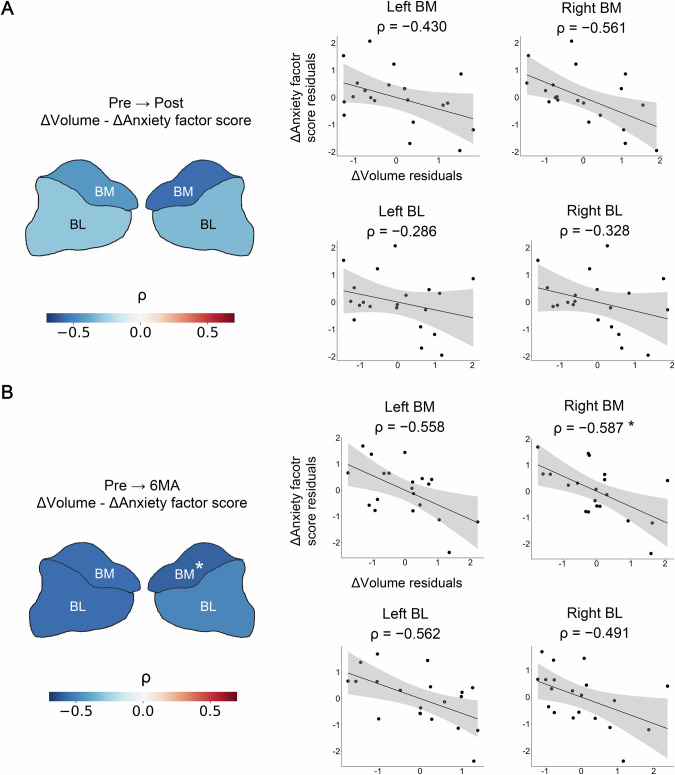
Association between changes in amygdala volume and anxiety factor score. **A** (Left) A heatmap depicting the partial correlation coefficients for Pre-to-Post changes. (Right) Scatterplots for Pre-to-Post changes. **B** (Left) A heatmap depicting the correlation coefficients for Pre-to-6MA changes. (Right) Scatterplots for Pre-to-6MA changes. BL Basolateral, BM Basomedial. *: *P* < 0.05 Bonferroni corrected.

The Pre-to-6MA volume change in the right BM subdivision exhibited a significant partial correlation with changes in the anxiety factor score (ρ = −0.587, 95% CI = [−0.817, −0.195], *P* = 0.010) (Fig. 4). None of the volume changes showed significant partial correlations with changes in the total HAMD-17 score (Supplementary Fig. S7).

### Association between baseline amygdala volumes and changes in anxiety severity

**Fig. 5:**
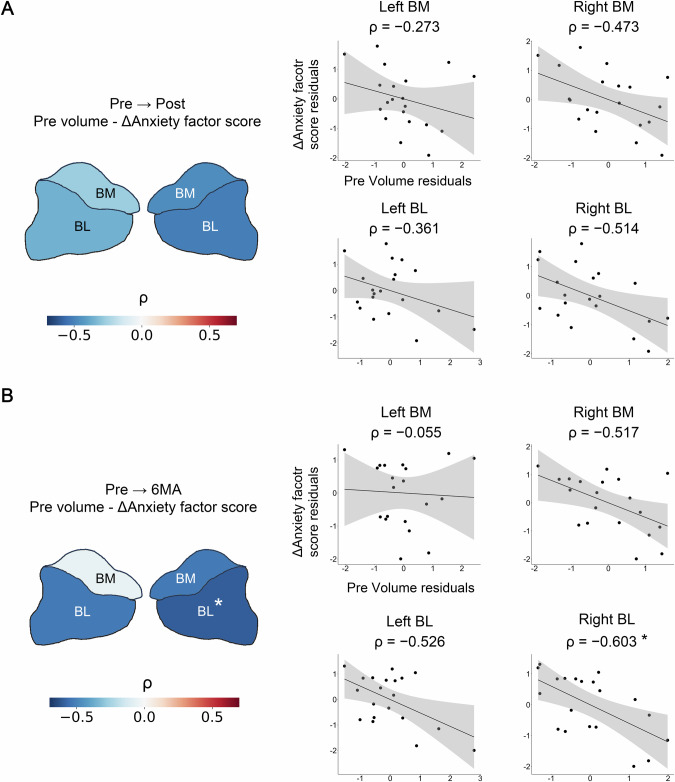
Association between baseline amygdala volumes and changes in anxiety factor score. **A** (Left) A heatmap depicting the partial correlation coefficients for Pre-to-Post changes. (Right) Scatterplots for Pre-to-Post changes. **B** (Left) A heatmap depicting the correlation coefficients for Pre-to-6MA changes. (Right) Scatterplots for Pre-to-6MA changes. BL Basolateral, BM Basomedial. *: *P* < 0.05 Bonferroni corrected.

Baseline right BL subdivision volumes exhibited a significant partial correlation with Pre-to-6MA changes in the anxiety factor score (ρ = −0.603, 95% CI = [−0.825, −0.219], *P* = 0.010) (Fig. 5). None of the baseline volumes showed significant partial correlations with changes in the total HAMD-17 score (Supplementary Fig. S7).

## Discussion

This study investigated short- and long-term volume changes in the amygdala subdivisions following ECT and other treatment modalities. Prior to treatment, both the BL and BM subdivisions of the right amygdala were smaller than those in the healthy control group. A post-treatment volume increase was observed only in the ECT group, followed by a subsequent decrease. At the 6-month follow-up, the amygdala subdivision volumes in the ECT group were significantly larger than those at the pre-treatment. Long-term volume changes in the right BM subdivision significantly correlated with improvements in anxiety symptoms. Finally, the baseline right BL subdivision volume indicated a long-term improvement in anxiety symptoms. These findings established a connection between the nuclei of the amygdala, ECT, and the manifestation of anxiety symptoms in depression, advancing our understanding of the therapeutic effects of ECT and the role of the amygdala in depression.

The current study included four different treatment modalities for depression and collected MRI and clinical data at three timepoints. Unlike previous studies, all treatment groups showed similar remission rates post-treatment [4]. This may be because the inclusion criteria for the ECT group necessitates treatment-resistant cases, resulting in a significantly more severe population compared to other treatment groups. Except for the medication group, age was also significantly different between ECT and non-ECT groups. Given these inter-group differences, the following discussion interprets the findings while considering these demographic and clinical variations.

In this study, we initially segmented amygdala nuclei using the FreeSurfer module [59]. Although this module offers highly reliable estimates for most nuclei, some exhibit relatively low reliability [60]. Furthermore, direct comparisons with prior partitioning methods [32, 61, 62] are not feasible, which poses an interpretability issue. To address this problem, we clustered the nine nuclei from the FreeSurfer output into three subdivisions based on their anatomical plausibility and proximity [30, 32, 59, 63]. We quantified the test-retest reliability using two-time-point data from a healthy control group, confirming enhanced reliability through clustering. This methodology, facilitated by collecting multi-time-point data from the healthy control group, significantly enhanced the reproducibility of the study. Additionally, the clustered subdivisions matched well with the cytoarchitectonically defined atlas and typical partitioning methods commonly used in animal research [30, 63], thereby improving the interpretability of the results. Notably, this clustering approach permits comparisons across different time points and individuals while accounting for individual variations in subregional positions.

This study, which included 155 patients with depression, is one of the most extensive investigations comparing amygdala volumes in depression. Pre-treatment right amygdala subdivision volumes in patients with depression were smaller than those in healthy controls, which is consistent with the results of a meta-analysis [69] and a previous study with a similar sample size [40]. For older cohorts aged 60 and above, there were no significant differences in baseline volume (Supplementary Table S5) while the effect sizes were larger than those reported in a previous meta-analysis (*d* = 0.208) [69]. There was no significant correlation between the pre-treatment volume and baseline HAMD-17, and anxiety factor scores. Previous research has suggested that sex differences in amygdala functionality may exist; therefore, the absence of clinical correlates may be attributed to demographic heterogeneity in patients, such as sex [70, 71]. Thus, future studies should consider an analytical design that can handle demographic heterogeneity, such as stratifying patients by sex, to reflect individual differences more accurately.

We observed unique treatment-induced volume changes in the amygdala subdivisions specifically associated with ECT, both in the short- and long-term. The transient increase in volume induced by ECT was followed by a decrease during the follow-up period, which is consistent with previous studies [51, 52]. Additionally, the effect sizes of the Pre-to-Post volume increases were comparable to those reported in a previous study [72]. The TMS group did not show significant volume changes in this study, although a previous study reported a significant increase in amygdala volume after 6 weeks of TMS sessions [73]. Therefore, further validation using a larger sample size is warranted. The medication group in this study did not show significant volume changes, whereas antidepressants were found to increase the amygdala volume [69, 74]. The medication group in this study primarily consisted of add-on cases, necessitating caution in interpreting the results due to the potential impact of past antidepressant use on baseline volume. Future studies focusing on treatment-naïve subjects may offer a more precise assessment of the influence of antidepressants on amygdala volume.

In this study, Pre-to-6MA volume increases in the right BM subdivision were significantly correlated with improvements in anxiety symptoms. The basomedial amygdala may play a distinct role in anxiety, as an animal study reported that the basomedial amygdala, but not the basolateral amygdala mediates the top-down control of anxiety [75]. Considering that activation of the basomedial amygdala ameliorates high-anxiety states as indicated in the previous study, an ECT-induced volume increase in basomedial amygdala may facilitate enhanced activation, leading to an improvement in anxiety states. Additionally, human studies have shown that the basolateral and basomedial amygdalae have different connectivity profiles [61, 76]. These findings underscore the importance of analyzing the amygdala at the nucleus level.

Furthermore, baseline volume of the right BL subdivision was identified as a potential biomarker for predicting improvements in anxiety symptoms. This finding is consistent with a previous study demonstrating that a larger amygdala volume predicted a greater improvement in overall depressive symptoms [25]. When all treatment groups were included, the baseline right BL subdivision volume was not predictive of anxiety improvement (Supplementary Fig. S9). Together with the results of the cross-sectional analysis in this study, this indicates that, while the right amygdala subdivisions are significantly smaller in patients with depression compared to healthy controls, a relatively larger volume in the right basolateral amygdala is associated with greater improvements in anxiety following ECT.

There is ongoing debate as to whether ECT-specific volume changes are merely by-products of seizure induction or if they contribute to treatment outcomes [77, 78]. One study suggested that ECT-induced volume increases were associated with regional electric field changes but not with antidepressant response, implying that these volume changes might not directly influence clinical outcomes [78]. Meanwhile, an animal study demonstrated electroconvulsive seizure-induced glial cell proliferation in the amygdala [79], suggesting that neuroplastic changes may contribute to the therapeutic effects of ECT. Although research on the amygdala is limited, studies focusing on the hippocampus have reported a broader range of neuroplastic changes, such as increased neuroplasticity markers and enhanced excitatory synaptic density [18, 19]. Furthermore, a previous study reported that the increase in hippocampal volume following ECT was not due to edema or angiogenesis [17], suggesting that distinct neuroplastic effects of ECT may result in volume increases.

Regarding the association between ECT-specific volume increases and treatment effects, previous research has reported conflicting findings [23–25]. Our findings add to this discourse by demonstrating that ECT-induced amygdala volume changes were not correlated with overall depressive symptoms (Supplementary Fig. S7). However, based on the hypothesis that clinical correlates of volume changes are specific to subregions and symptoms, we demonstrated significant associations between amygdala subdivision volumes and improvements in anxiety. These findings emphasize the importance of examining treatment effects at the subregion and symptom levels. A study focusing on healthy participants found that functional connectivity of the amygdala nuclei could predict subclinical mental health dimensions [80]. This finding highlights the importance of exploring the functional connectivity of amygdala subdivisions and their relationship with multiple symptom dimensions in patients with depression, thereby advancing our understanding of the underlying mechanisms of depression.

Interestingly, the TMS group showed significant associations between long-term changes in the left amygdala subdivisions and anxiety improvements, albeit showing insignificant volume changes (Supplementary Fig. S8). Furthermore, smaller baseline left BL volumes significantly correlated with long-term anxiety symptom improvements (Supplementary Fig. S8). Despite the inconsistent laterality, both the ECT and TMS groups exhibited that larger changes in amygdala subdivision volumes are associated with greater improvements in anxiety. In contrast, when examining the association between baseline amygdala volume and anxiety improvement, these groups showed opposite correlation coefficients; larger baseline right BL volumes correlated with greater anxiety improvement in the ECT group, while smaller baseline left BL volumes correlated with greater anxiety improvement in the TMS group. While the correlation directions differ between treatment modalities, these findings are consistent with previous studies. Doesschate et al. [25] reported larger baseline amygdala volumes as predictive of greater improvement in depressive symptoms following ECT, and Furtado et al. [81] reported smaller baseline right amygdala volume as predictive of greater improvement in depressive symptoms following TMS. The inconsistency in correlation directions between ECT and TMS may reflect their distinct mechanisms of action and warrants further investigation.

This study has some limitations, including its observational design, which resulted in differences in severity and demographic characteristics between the treatment groups, as well as variations in the ECT treatment protocols. Since the ECT group consisted of treatment-resistant cases, which differed from the populations in the other treatment groups, caution is advised when interpreting the specificity of the volume changes associated with ECT. In addition, the test-retest reliability of the CM subdivision remained low even after clustering. Given that the centromedial amygdala is considered a key region in affective and behavioral processes [30, 35], future research should focus on identifying the centromedial amygdala using ultra-high-resolution MRI images or deep learning-based automated segmentation [53, 82]. Finally, it is desirable for future studies to validate the current findings using a larger sample size of the ECT group.

In summary, this study discovered ECT-specific amygdala volume changes at the subdivision level over both short- and long-term periods. Additionally, this study identified correlations between right basomedial amygdala volumes and improvements in anxiety symptoms. Furthermore, baseline volumes in the basolateral amygdala significantly correlated with improvements in anxiety. These findings provide insight into the amygdala as a potential biomarker for depression treatment and highlight the importance of examining treatment outcomes at fine-grained levels, considering localized brain regions and specific symptom dimensions.

## Supplementary information

supplementary_information

## Data Availability

Data supporting the findings of this study are available from the corresponding author upon request. The data are not publicly available because they contain information that could compromise the privacy of the research participants.
