# Supplementary material for: Electroconvulsive therapy-specific volume changes in nuclei of the amygdala and their relationship to long-term anxiety improvement in depression
Source: Mol Psychiatry. 2024 Dec 16;30(6):2653–64. doi: 10.1038/s41380-024-02874-1 (PMC12092276; doi:10.1038/s41380-024-02874-1)
Supplement: Supplementary file 1 — supplementary_information [file 41380_2024_2874_MOESM1_ESM.docx]

**Supplementary Information**

**Supplementary Methods**

**Electroconvulsive therapy**

To determine the stimulus intensity, the half-age stimulation strategy was used for patients receiving bilateral stimulation, and the titration stimulation strategy was used for those receiving right unilateral stimulation. The Low 0.5 program was used to determine the pulse width, except for one patient who switched from right unilateral to bifrontal stimulation, for whom Low 0.25 was used for the right unilateral stimulation.

**Anxiety factor score**

Factor loadings reported by Shafer et al. (2006) were used to calculate the anxiety factor score. The anxiety factor score was defined as a sum of the HAMD-17 subscales weighted by the factor loadings (Depression mood: 0.17, Guilt: 0.25, Suicide: 0.01, Insomnia initial: 0.07, Insomnia middle: 0.07, Insomnia delayed: 0.05, Work and interests: 0.10, Retardation: 0.02, Agitation: 0.84, Anxiety psychic: 0.85, Anxiety somatic: 0.80, Gastrointestinal: 0.20, General somatic: 0.05, Libido loss: −0.03, Hypochondriasis: 0.45, Weight loss; 0.25, Insight loss: 0.35).

**Supplementary Results**

**Intervals of ECT sessions**

Among the ECT group (N = 20), seven participants received ECT sessions at a once-weekly interval due to physical illnesses (four participants received two sessions and three participants received one session at a once-weekly interval). All sessions for the remaining participants were conducted at a twice-weekly interval.

**ECT sessions during follow-up period**

One patient received eight additional maintenance ECT sessions twice a week during the follow-up period. None of the other patients took maintenance ECT sessions during the follow-up period.

**Medication changes during follow-up period**

Among the medication group (N = 52), 22 patients reduced the number of medications, 5 patients underwent a switch, and 16 patients underwent add-on therapy during the follow-up period. Nine patients continued the same medication with those during the pre-post period.

**Interaction effects between age and group on pre-treatment volume comparison**

There were no interaction effects between age and group (depression, healthy controls) on pre-treatment amygdala subdivision volumes (Left BL: *F*[1, 297] = 0.02, *P* = .881; Left BM: *F*[1, 297] = 0.01, *P* = .940; Right BL: *F*[1, 297] = 0.27, *P* = .601; Right BM: *F*[1, 297] = 0.0, *P* = .955).

**Interaction effect between scanning sites and TMS sessions**

The number of TMS sessions for each scanning site were as follows: Site 1: 26.8 (2.9); Site 3: 24.5 (2.8); Site 4: 24.5 (2.7) (Supplementary Tables S2-3). The interaction effect between the scanning sites and number of TMS sessions was not significant (*F*[2, 17] = 3.43, *p* = .056).

**Supplementary Tables**

**Supplementary Table 1. Parameters of electroconvulsive therapy.**

**Supplementary Table 2. Parameters of transcranial magnetic stimulation.**

**Supplementary Table 3. MRI parameters and the number of subjects.**

**Supplementary Table 4. Baseline amygdala volumes.**

**Supplementary Table 5. Baseline amygdala volumes for age-stratified subjects.**

**Supplementary Table 6. Volume changes in amygdala subdivisions.**

**Supplementary Table 7. Patients age-matched to the ECT group.**

**Supplementary Table 8. Patients with top 25% anxiety factor scores.**

**Supplementary Table 9. The number of ECT sessions and volume changes.**

**Supplementary Figures**

**Supplementary Figure 1. Test-retest reliability of volume measurements.**

**Supplementary Figure 2. Longitudinal volume changes.**

**Supplementary Figure 3. Heatmaps depicting mean percentage volume changes.**

**Supplementary Figure 4. Volume changes with patients age-matched to the ECT group.**

**Supplementary Figure 5. Volume changes with top 25% anxiety factor scores.**

**Supplementary Figure 6. Volume changes for ECT-remitters and non-remitters**

**Supplementary Figure 7. Comparison of HAMD-17 and anxiety factor score.**

**Supplementary Figure 8. Correlations with anxiety for the TMS group.**

**Supplementary Figure 9. Right basolateral amygdala volume and anxiety prediction. Supplementary Table 1. Parameters of electroconvulsive therapy (ECT).**

| Variable | Mean (SD) | Range |
| --- | --- | --- |
| Number of ECT sessions | 11.1 (3.2) | 8–19 |
| Number of days between first and last ECT session | 36.2 (15.8) | 18–72 |
| Stimulus power (mC) | 242.5 (143.1) | - |
| EEG seizure length (sec) | 48.0 (24.0) | - |
| Postictal suppression index (%) | 72.4 (22.5) | - |

**Supplementary Table 2. Parameters of transcranial magnetic stimulation (TMS).**

| Variable |  |
| --- | --- |
| Number of TMS sessions, mean (SD) | 26.8 (3.4) |
| Number of TMS sessions for Site 1, mean (SD) | 26.8 (2.9) |
| Number of TMS sessions for Site 3, mean (SD) | 24.5 (2.8) |
| Number of TMS sessions for Site 4, mean (SD) | 24.5 (2.7) |
| Number of days between first and last TMS session, range | 20–50 |
| Number of pulses per session | 3000 |
| Duty cycle on (sec) | 4 |
| Duty cycle off (sec) | 26 |
| Pulse frequency (Hz) | 10 |
| Power (% resting motor threshold), range | 80–120 |

**Supplementary Table 3. MRI parameters and the number of subjects for each condition.** ECT: Electroconvulsive therapy; CBT: Cognitive behavioral therapy; TMS: Transcranial magnetic stimulation; HC: Healthy controls.

| Variable | Site 1 | | Site 2 | | Site 3 | Site 4 |
| --- | --- | --- | --- | --- | --- | --- |
|  | GE  Discovery MR750 | SIGNA HDxt | MAGNETOM Verio | MAGNETOM Skyra | MAGNETOM Skyra fit | MAGNETOM Skyra |
| Repetition time (ms) | 6.77 | 6.41 | 2500 | | | |
| Echo time (ms) | 3.01 | 2.78 | 2.18 | | | |
| Flip angle (°) | 8 | 8 | 8 | | | |
| Number of slices | 200 | 200 | 224 | | | |
| Field of view | 230×230 | 230×230 | 240×256 | | | |
| Matrix size | 256×256 | 256×256 | 300×320 | | | |
| Spatial resolution (mm) | 0.9×0.9×1.0 | 0.9×0.9×1.0 | 0.8×0.8×0.8 | | | |
| Number of participants | | | | | | |
| ECT | 0 | 4 | 0 | 5 | 11 | 0 |
| Medication | 13 | 7 | 16 | 0 | 16 | 0 |
| CBT | 20 | 1 | 31 | 0 | 11 | 0 |
| TMS | 0 | 8 | 0 | 0 | 6 | 6 |
| HC | 40 | 10 | 48 | 6 | 43 | 0 |

**Supplementary Table 4. Baseline amygdala volumes, mean (SD).** HC: Healthy controls. *: *P* < 0.05 Bonferroni corrected.

|  | Left | | | Right | | |
| --- | --- | --- | --- | --- | --- | --- |
|  | Depression | HC | *p*-value | Depression | HC | *p-*value |
| **Basolateral** | 1217.8 (144.6) | 1225.7 (127.8) | .304 | 1258.7 (150.8) | 1288.0 (130.1) | .009* |
| **Basomedial** | 420.8  (57.7) | 428.8  (54.9) | .069 | 457.2  (60.9) | 470.7  (58.7) | .005* |

**Supplementary Table 5.** **Baseline amygdala volumes for age-stratified participants.** In stratifying the participants into those aged 60 and above (Depression: N = 26, age mean (SD): 69.3 (7.0); HC: N = 16, age mean (SD): 67.6 (4.9) ) and those below 60 (Depression: N = 129, age mean (SD): 39.4 (11.5); HC: N = 131, age mean (SD): 38.9 (9.9) ), right BL and BM volumes in patients with depression remained significantly smaller compared to healthy controls in the group aged below 60, while no significant differences were found in the group aged 60 and above.

|  | Age ≥ 60 | | | | Age < 60 | | | |
| --- | --- | --- | --- | --- | --- | --- | --- | --- |
|  | Depression  (N = 26) | HC  (N = 16) | Cohen’s *d* | *p*-value | Depression  (N = 129) | HC  (N = 131) | Cohen’s *d* | *p-*value |
| **Left**  **Basolateral** | 1071.2 (160.5) | 1144.2 (106.8) | .51 | .370 | 1238.4 (121.3) | 1235.3 (121.7) | .02 | .608 |
| **Left**  **Basomedial** | 357.9  (54.5) | 384.2  (47.8) | .50 | .276 | 430.4  (49.3) | 434.8  (50.4) | .09 | .165 |
| **Right**  **Basolateral** | 1124.6  (136.4) | 1171.6 (116.2) | .36 | .755 | 1277.9 (132.2) | 1302.6 (120.1) | .20 | .009* |
| **Right**  **Basomedial** | 392.6  (51.0) | 411.9  (48.8) | .38 | .556 | 467.2  (50.1) | 478.6  (52.0) | .22 | .005* |

**Supplementary Table 6. Longitudinal changes in amygdala subdivision volumes, mean (SD).** ECT: Electroconvulsive therapy; CBT: Cognitive behavioral therapy; TMS: Transcranial magnetic stimulation; HC: Healthy controls.

|  |  |  | **ECT** | **Medication** | **CBT** | **TMS** | **HC** |
| --- | --- | --- | --- | --- | --- | --- | --- |
| **N** |  |  | 20 | 52 | 63 | 20 | 147 |
| **Left** | **Basolateral** | Pre | 1136.0 (133.9) | 1236.2 (170.9) | 1211.2 (120.8) | 1232.6 (139.6) | 1225.7 (127.8) |
|  |  | Post | 1203.0 (145.6) | 1239.4 (174.4) | 1212.8 (113.1) | 1231.1 (142.5) | 1226.4 (130.0) |
|  |  | 6MA | 1166.2 (144.1) | 1237.9 (168.7) | 1214.6 (122.8) | 1231.5 (146.1) | - |
|  | **Basomedial** | Pre | 385.7 (60.5) | 422.0 (64.7) | 424.1 (50.4) | 429.9 (55.0) | 428.8 (54.9) |
|  |  | Post | 411.6 (65.9) | 424.7 (66.4) | 422.0 (47.1) | 428.2 (55.8) | 429.5 (54.1) |
|  |  | 6MA | 396.8 (65.5) | 425.3 (65.6) | 423.3 (48.2) | 426.5 (55.7) | - |
| **Right** | **Basolateral** | Pre | 1185.9 (157.1) | 1274.4 (163.4) | 1255.6 (139.7) | 1273.4 (110.3) | 1288.0 (130.1) |
|  |  | Post | 1259.4 (170.0) | 1274.6 (173.0) | 1248.1 (133.1) | 1279.7 (126.6) | 1283.7 (143.6) |
|  |  | 6MA | 1215.7 (170.2) | 1269.2 (168.9) | 1259.8 (145.5) | 1277.4 (126.3) | - |
|  | **Basomedial** | Pre | 426.7 (73.7) | 459.3 (62.4) | 457.2 (51.8) | 466.7 (53.4) | 470.7 (58.7) |
|  |  | Post | 452.1 (82.1) | 459.2 (66.4) | 457.2 (50.9) | 464.6 (56.5) | 469.0 (59.8) |
|  |  | 6MA | 436.3 (79.1) | 459.2 (66.8) | 460.9 (53.9) | 465.6 (54.4) | - |

**Supplementary Table 7. Clinical characteristics of patients age-matched to the ECT group.** Age was matched using the caliper width of three years (equivalent to 0.2 SD of the depression group’s age) and did not significantly differ between treatment groups. Longitudinal volume changes of these subjects are shown in Supplementary Figure S4. ECT: Electroconvulsive therapy; CBT: Cognitive behavioral therapy; TMS: Transcranial magnetic stimulation; HC: Healthy controls.

|  | **ECT**  **(all subjects)** | **Medication** | **CBT** | **TMS** |
| --- | --- | --- | --- | --- |
| N | 20 | 20 | 15 | 14 |
| Sex (M/F) | 7/13 | 8/12 | 7/8 | 7/7 |
| Age, mean (SD), yrs | 55.1 (17.1) | 54.7 (16.7) | 48.9 (14.8) | 47.8 (14.8) |
| Pre HAMD-17, mean (SD) | 25.0 (6.3) | 15.9 (5.3) | 15.7 (5.5) | 16.8 (4.6) |
| Post HAMD-17, mean (SD) | 7.8 (5.6) | 7.4 (5.5) | 9.3 (7.4) | 10.9 (7.1) |
| 6MA HAMD-17, mean (SD) | 6.7 (7.2) | 6.2 (7.1) | 9.3 (6.0) | 10.3 (6.2) |
| Pre anxiety factor score, mean (SD) | 6.15 (2.22) | 4.15 (1.87) | 3.69 (1.35) | 3.81 (1.99) |
| Post anxiety factor score, mean (SD) | 1.72 (1.38) | 2.00 (1.75) | 2.24 (2.11) | 2.31 (1.25) |
| 6MA anxiety factor score, mean (SD) | 1.36 (1.61) | 1.83 (1.96) | 1.97 (1.78) | 2.39 (2.03) |

**Supplementary Table 8. Clinical characteristics of patients with top 25% anxiety factor scores.** We analyzed subsets from the other treatment groups to adjust for differences in baseline anxiety factor scores. The baseline anxiety factor scores did not significantly differ between treatment groups. Longitudinal volume changes of these subsets are shown in Supplementary Figure S5. ECT: Electroconvulsive therapy; CBT: Cognitive behavioral therapy; TMS: Transcranial magnetic stimulation; HC: Healthy controls.

|  | **ECT**  **(all subjects)** | **Medication** | **CBT** | **TMS** |
| --- | --- | --- | --- | --- |
| N | 20 | 13 | 15 | 5 |
| Sex (M/F) | 7/13 | 6/7 | 6/9 | 4/1 |
| Age, mean (SD), yrs | 55.1 (17.1) | 50.8 (15.9) | 36.1 (12.8) | 50.0 (15.1) |
| Pre HAMD-17, mean (SD) | 25.0 (6.3) | 22.0 (5.6) | 19.5 (3.8) | 21.4 (2.3) |
| Post HAMD-17, mean (SD) | 7.8 (5.6) | 10.6 (9.0) | 10.5 (4.9) | 15.8 (7.4) |
| 6MA HAMD-17, mean (SD) | 6.7 (7.2) | 9.9 (10.4) | 8.9 (4.7) | 13.6 (7.3) |
| Pre anxiety factor score, mean (SD) | 6.15 (2.22) | 6.15 (1.44) | 5.74 (0.87) | 5.81 (1.21) |
| Post anxiety factor score, mean (SD) | 1.72 (1.38) | 3.16 (2.72) | 2.95 (1.75) | 2.97 (1.02) |
| 6MA anxiety factor score, mean (SD) | 1.36 (1.61) | 2.93 (3.06) | 2.59 (2.03) | 3.57 (2.51) |

**Supplementary Table 9. Associations between the number of ECT sessions and amygdala volume changes.** Spearman’s rank correlation analysis was performed to examine the associations between the number of ECT sessions and amygdala volume changes. There was no association between the number of sessions and volume changes. The mean, SD, and range of the number of ECT sessions are provided in Supplementary Table 1. BL: Basolateral; BM: Basomedial.

|  |  | Left BL | Left BM | Right BL | Right BM |
| --- | --- | --- | --- | --- | --- |
| Pre - Post | Spearman’s ρ | .10 | .00 | .08 | .15 |
|  | p-value | .66 | .99 | .74 | .53 |
| Pre - 6MA | Spearman’s ρ | .17 | .36 | .12 | .09 |
|  | p-value | .49 | .11 | .63 | .71 |


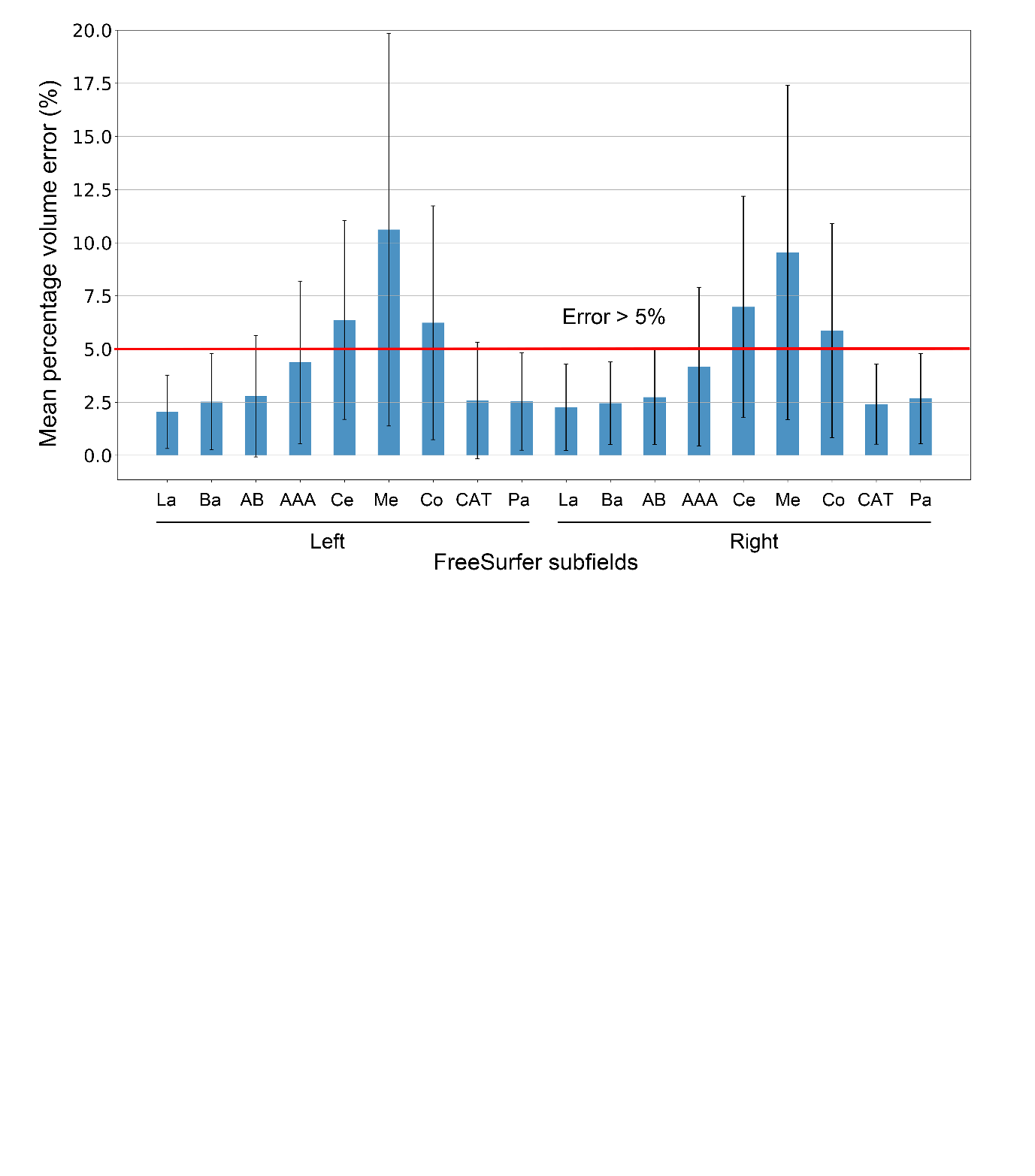


**Supplementary Figure 1. Test-retest reliability of the volume measurements of amygdala subfields generated by FreeSurfer.** Test-retest reliability was quantified as the mean percentage volume error of healthy controls (see Materials and Methods). We established that less than 5% of the mean percentage volume error is sufficient to conduct longitudinal comparison (Quattrini et al., 2020). La: Lateral; Ba: Basal; AB: Accessory basal; AAA; Anterior amygdala area; Ce: Central; Me: Medial; Co: Cortical; CAT; Cortico-amygdaloid transition area; Pa: Paralaminar.


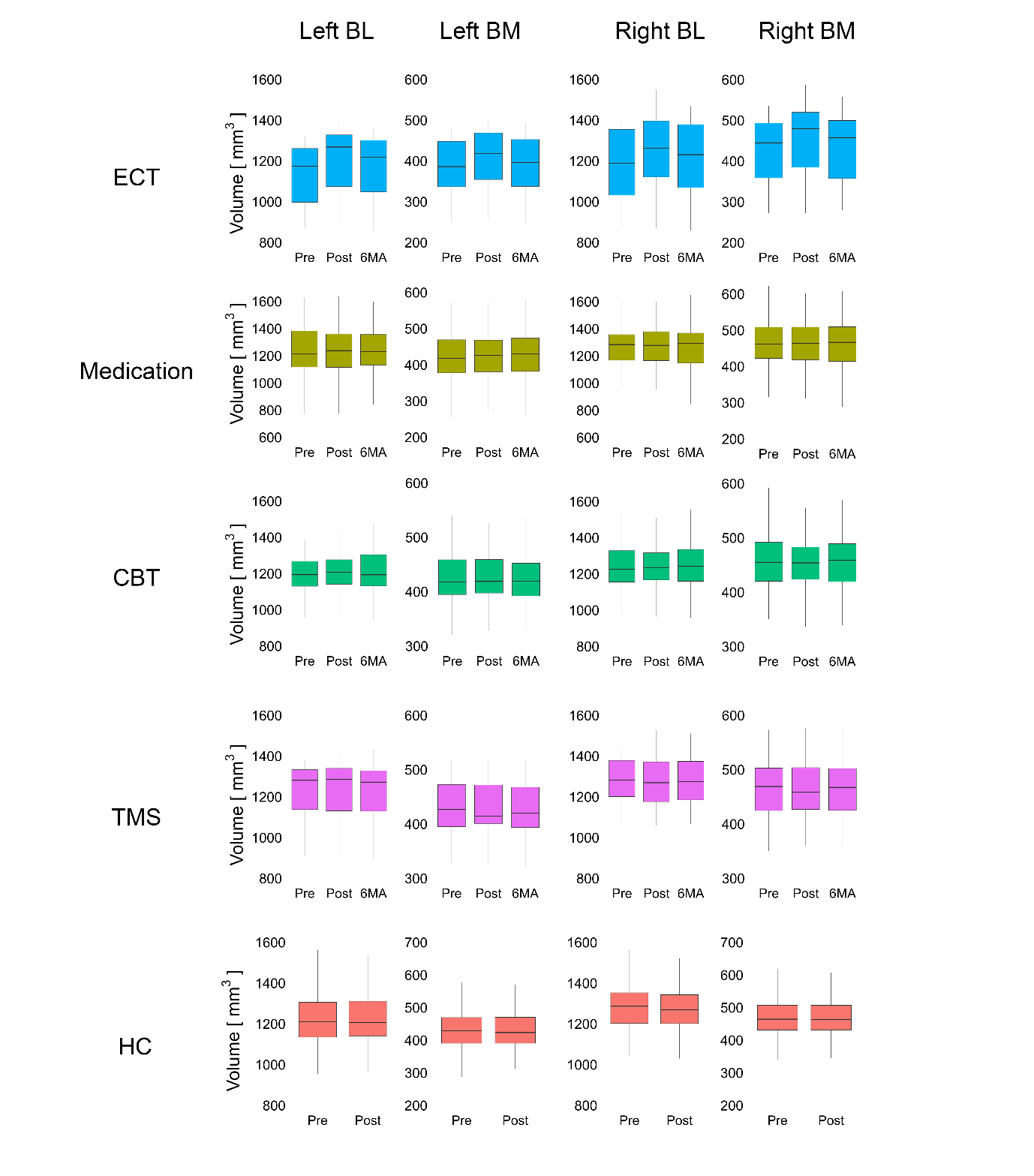


**Supplementary Figure 2. Longitudinal volume changes in the amygdala subdivisions for each group.** ECT: Electroconvulsive therapy; CBT: Cognitive behavioral therapy; TMS: Transcranial magnetic stimulation; HC: Healthy controls; BL: Basolateral; BM: Basomedial.


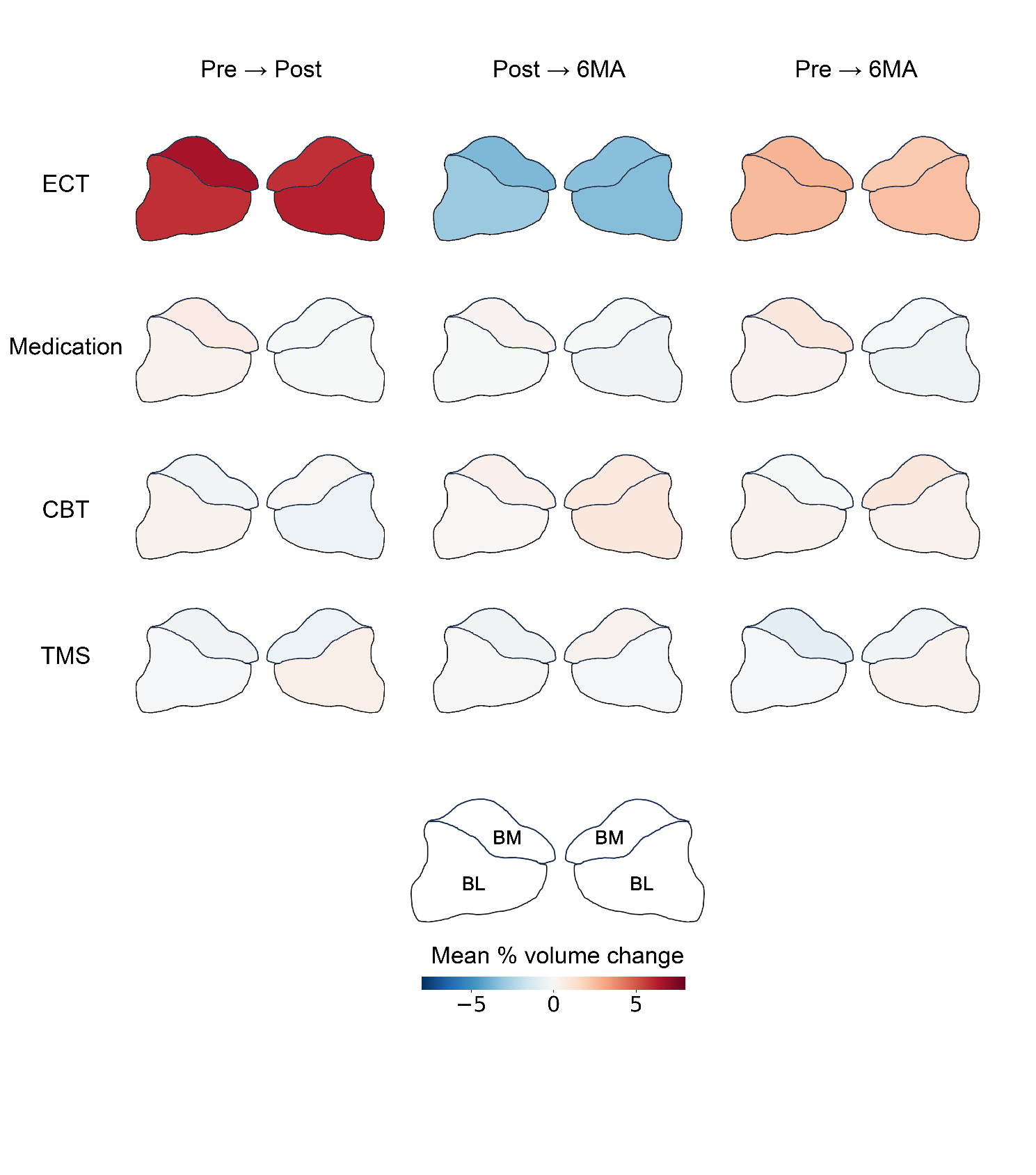


**Supplementary Figure 3. Heatmaps depicting mean percentage volume changes for each group.** ECT: Electroconvulsive therapy; CBT: Cognitive behavioral therapy; TMS: Transcranial magnetic stimulation; BL: Basolateral; BM: Basomedial.


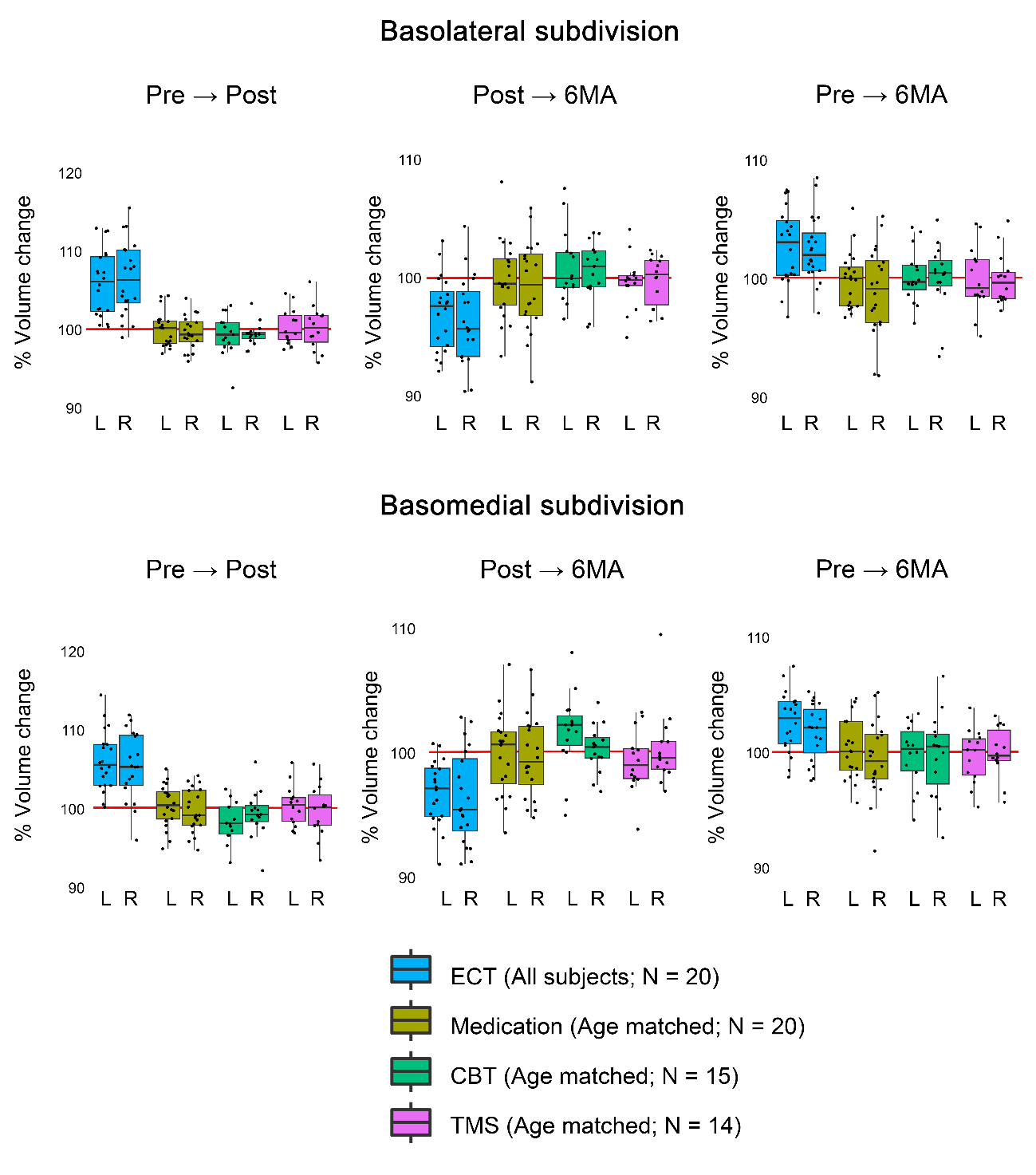


**Supplementary Figure 4. Percentage volume changes of the amygdala subdivisions for age-matched patients to the ECT group.** To examine whether age differences confounded volume changes, we selected subsets from the other treatment groups to match the age to those of the ECT group using a caliper matching method with a width of three years (Austin et al., 2011). The clinical characteristics of these subsets are shown in Supplementary Table S7. ECT: Electroconvulsive therapy; CBT: Cognitive behavioral therapy; TMS: Transcranial magnetic stimulation.


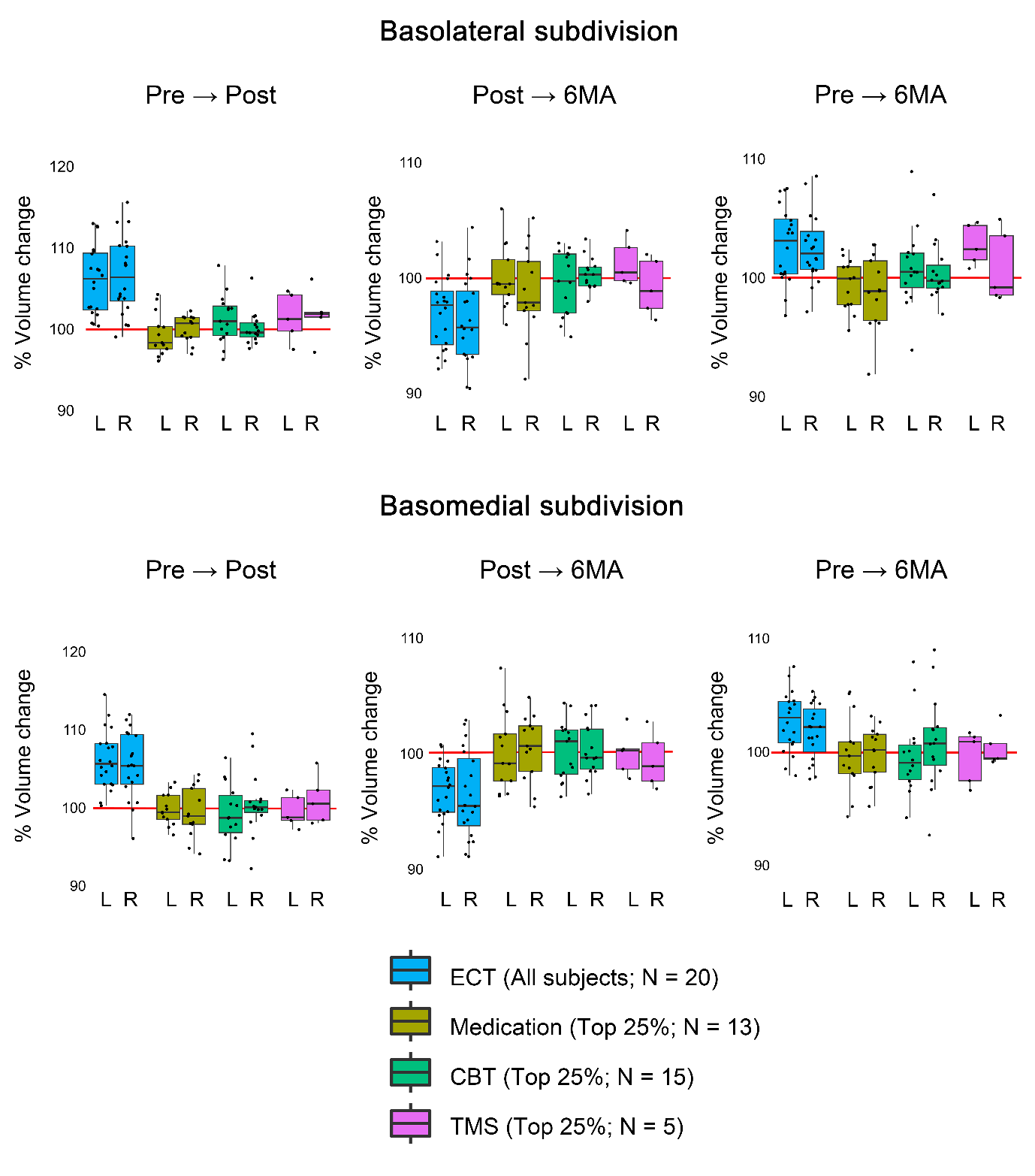


**Supplementary Figure 5.** **Percentage volume changes of the amygdala subdivisions for patients with top 25% anxiety factor score.** To examine whether baseline clinical scores confounded volume changes, we selected subsets from the other treatment groups to match the baseline anxiety factor score to those of the ECT group. Clinical characteristics of these subsets are shown in Supplementary Table S8. ECT: Electroconvulsive therapy; CBT: Cognitive behavioral therapy; TMS: Transcranial magnetic stimulation.


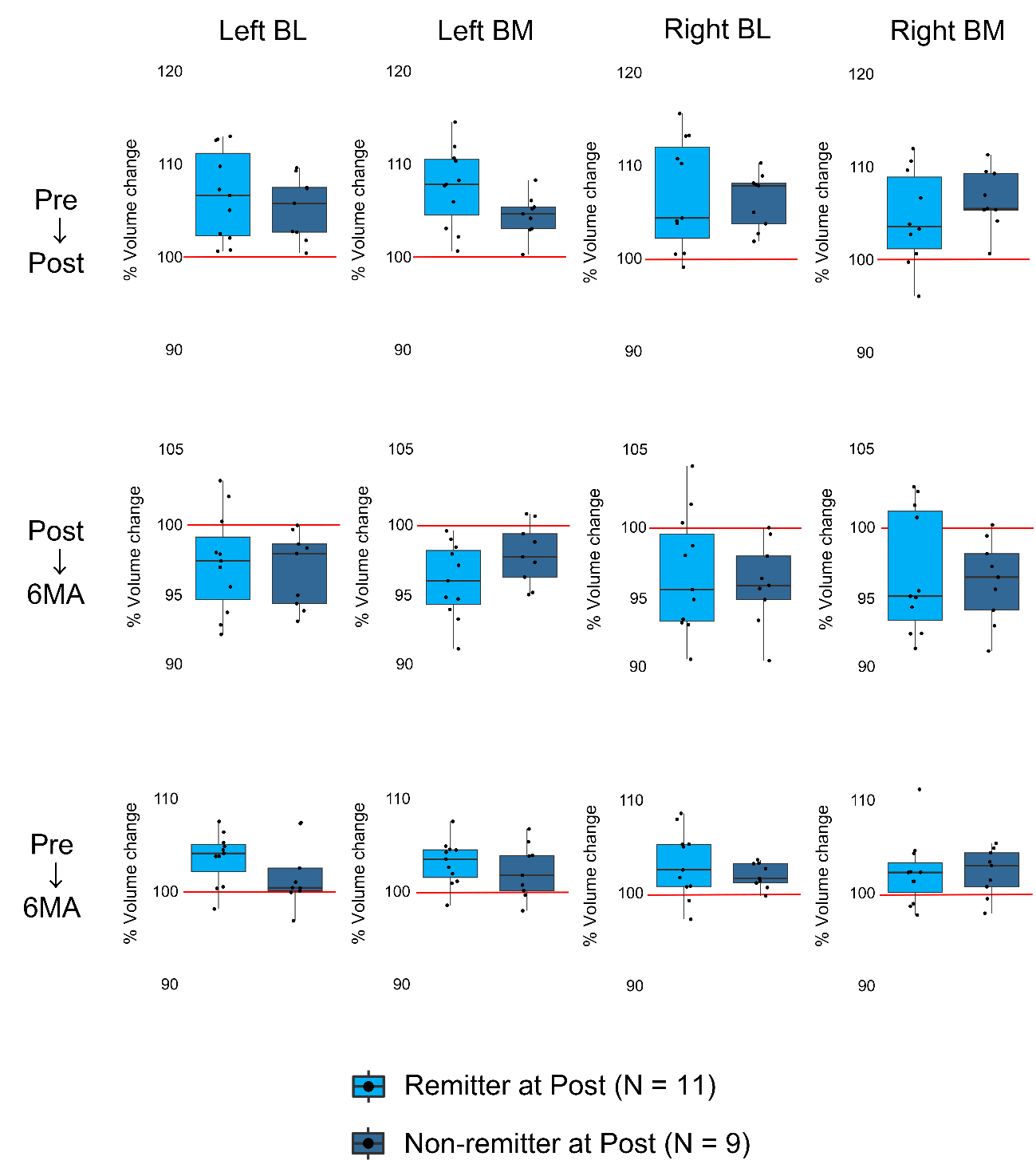


**Supplementary Figure 6. Volume changes for ECT remitters (N = 11) and non-remitters (N = 9).** Remission was defined as HAMD-17 <8 at the post-treatment assessment. BL: Basolateral; BM: Basomedial.

**
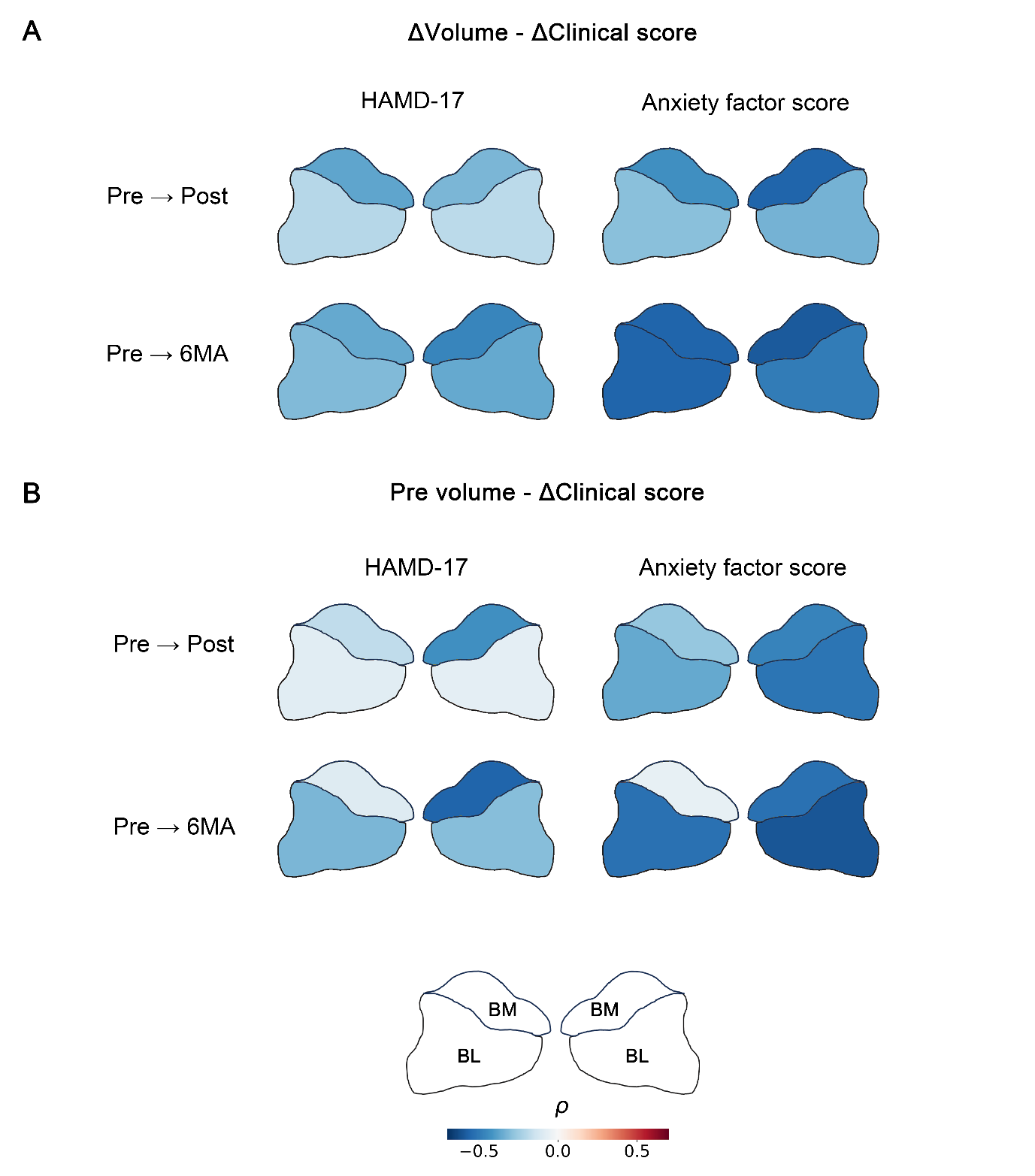
**

**Supplementary Figure 7. Partial correlation coefficients with volume measurements for the total HAMD-17 and anxiety factor score.** (**A**) Heatmaps depicting partial correlation coefficients between changes in volumes and clinical scores. (**B**) Heatmaps depicting partial correlation coefficients between baseline volumes and changes in clinical scores. Amygdala subdivision volumes exhibited greater correlation coefficients with anxiety factor scores than with HAMD-17. BL: Basolateral; BM: Basomedial.


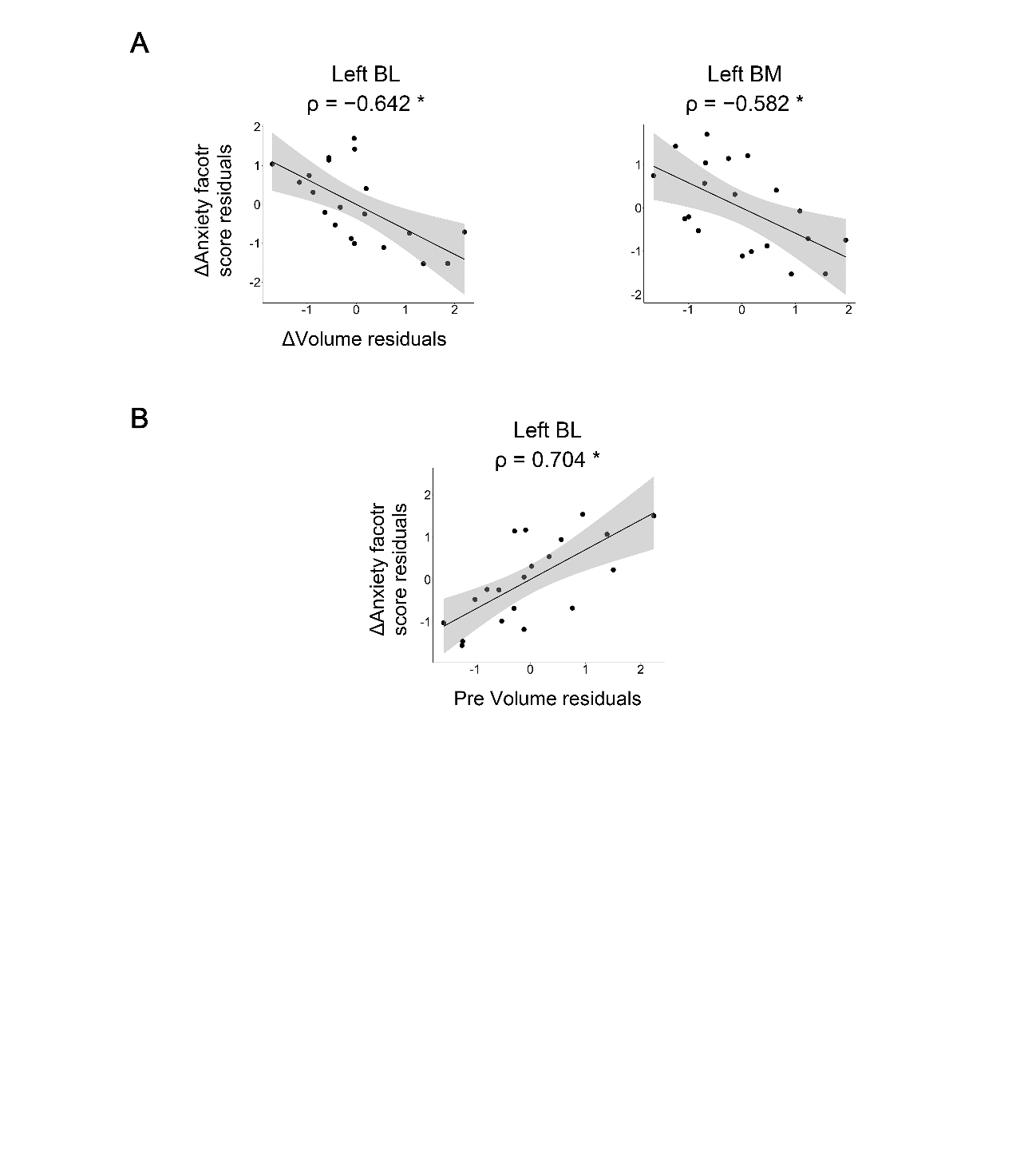


**Supplementary Figure 8. Significant partial correlations between amygdala subdivision volumes and Pre-to-6MA changes in the anxiety factor score for the TMS group.** (**A**) Scatterplots for partial correlation between Pre-to-6MA changes in the amygdala subdivision volumes and anxiety factor score (left BL: ρ = −.642, 95% CI = [−.845, −.279], *P* = .004; left BM: ρ = −.582, 95% CI = [−.815, −.187], *P* = .011). (**B)** Scatterplots for partial correlation between baseline left BL volumes and Pre-to-6MA changes in the anxiety factor score (Left BL: ρ = .704, 95% CI = [.380, .874], *P* = .002). BL: Basolateral; BM: Basomedial. *: *P* < 0.05 Bonferroni corrected.


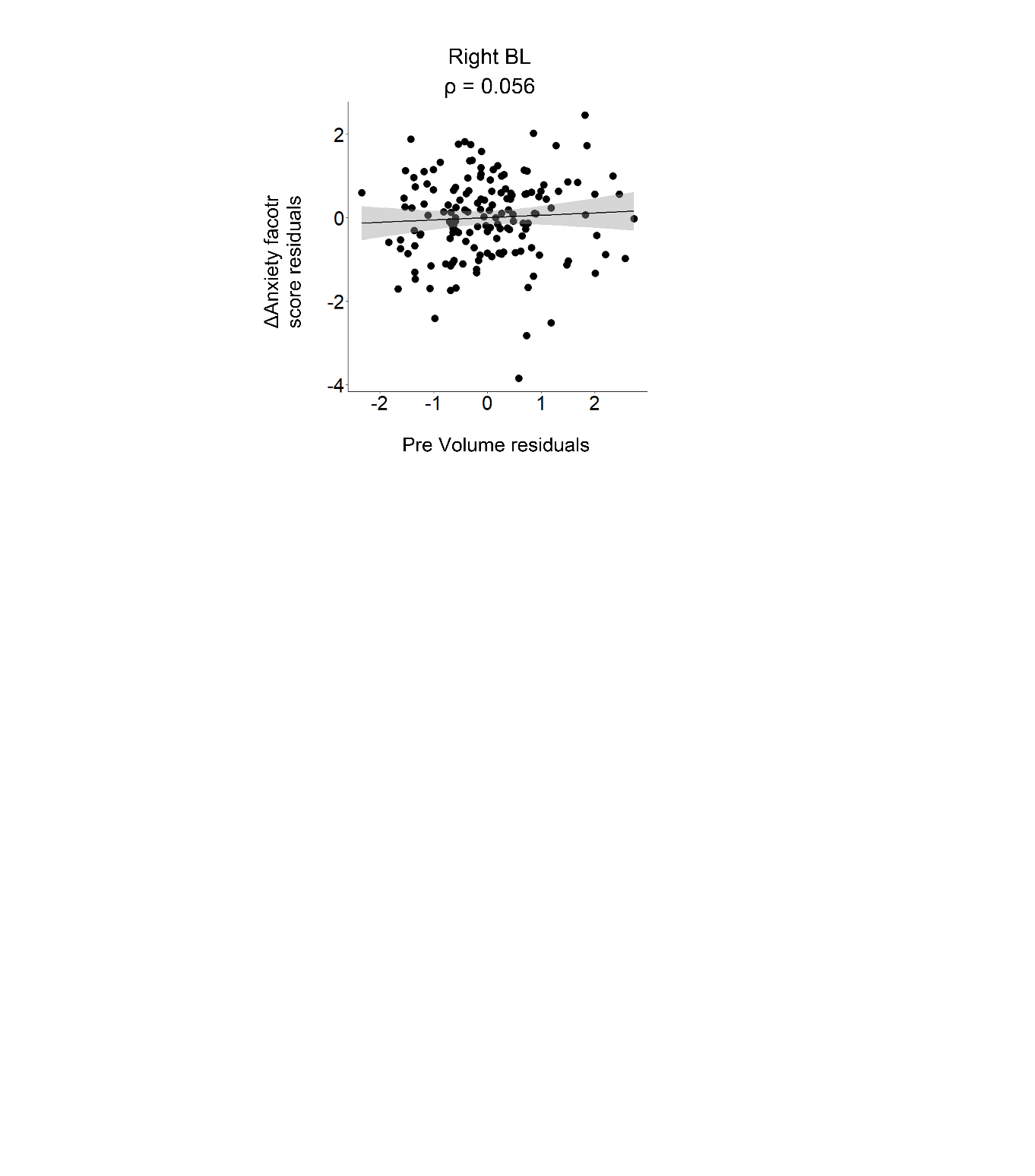


**Supplementary Figure 9. Association between baseline right basolateral amygdala volumes and Pre-to-6MA changes in anxiety factor scores for all patients with depression**. BL: Basolateral.

## **References**

Austin PC. Optimal caliper widths for propensity‐score matching when estimating differences in means and differences in proportions in observational studies. *Pharm Stat*. 2011;10:150–161.

Quattrini G, Pievani M, Jovicich J, Aiello M, Bargalló N, Barkhof F, et al. Amygdalar nuclei and hippocampal subfields on MRI: Test-retest reliability of automated volumetry across different MRI sites and vendors. *Neuroimage*. 2020;218:116932.
